# Stereotactic Radiotherapy Followed by Surgical Stabilization Within 24 h for Unstable Spinal Metastases; A Stage I/IIa Study According to the IDEAL Framework

**DOI:** 10.3389/fonc.2018.00626

**Published:** 2018-12-20

**Authors:** Anne L. Versteeg, Joanne M. van der Velden, Jochem Hes, Wietse Eppinga, Nicolien Kasperts, Helena M. Verkooijen, F. C. Oner, Enrica Seravalli, Jorrit-Jan Verlaan

**Affiliations:** ^1^Department of Orthopedics, University Medical Center Utrecht, Utrecht, Netherlands; ^2^Department of Radiation Oncology, University Medical Center Utrecht, Utrecht, Netherlands

**Keywords:** spinal metastases, SBRT, surgery, safety, phase I/II

## Abstract

**Background:** Routine treatment for unstable spinal metastases consists of surgical stabilization followed by external beam radiotherapy (EBRT) or stereotactic body radiotherapy (SBRT) after a minimum of 1–2 weeks to allow for initial wound healing. Although routine treatment, there are several downsides. First, radiotherapy induced pain relief is delayed by the time interval required for wound healing. Second, EBRT often requires multiple hospital visits and only 60% of the patients experience pain relief. Third, spinal implants cause imaging artifacts hindering SBRT treatment planning and delivery. Reversing the order of surgery and radiotherapy, with dose sparing of the surgical area by SBRT, could overcome these disadvantages and by eliminating the interval between the two treatments, recovery, and palliation may occur earlier.

**Design:** The safety of SBRT followed by surgical stabilization within 24 h for the treatment of unstable spinal metastases was investigated. Safety was evaluated using the Common-Toxicity-Criteria-Adverse-Events-4.0, with the occurrence of wound complications within 90-days being the primary concern.

**Results:** Between June-2015 and January-2017, 13 patients underwent SBRT followed by surgical stabilization for unstable spinal metastases. The median time between SBRT and surgery was 17-h (IQR 5–19). None of the patients experienced wound complications. Improvements in pain and quality of life were observed over time for all patients.

**Conclusion:** SBRT followed by surgical stabilization within 24 h for the treatment of unstable spinal metastases is safe. Palliation may be experienced earlier and with both treatments being performed in one hospital admission the treatment burden decreases.

## Introduction

More than half of the newly diagnosed cancer patients suffer from a tumor that frequently metastasizes to the bones (1), with the spine being the most frequent site (2). In addition to pain, spinal metastases can cause mechanical instability and/or spinal cord compression. The combination of surgery and radiotherapy is increasingly being used in the management of patients with symptomatic spinal metastases. Surgery is used for stabilization of the spinal column and/or to decompress neurological structures while post-operative radiotherapy aims for additional pain relief and local tumor control. This approach has shown to be effective to reduce pain and maintain or improve functional status and quality of life (3, 4).

While conventional external beam radiation therapy (EBRT) has been the mainstay of post-operative adjuvant radiotherapy, there are several concerns regarding its use in patients with spinal metastases. Precise targeting is limited with conventional EBRT and, as a consequence, the tolerance of the spinal cord limits the radiation dose to the vertebral body with pain relief achieved in only 60% of patients and local tumor control in only 30% of the patients after 1 year (5–7). Furthermore, to reach adequate radiation doses to the metastasis, hotspots of >120% of the prescribed dose are common in the subcutaneous tissues, which impairs wound healing (8). As such, a minimum time interval of 1–2 weeks between surgery and EBRT is considered necessary but thereby also delays radiotherapy-induced pain relief (8).

Stereotactic body radiotherapy (SBRT) allows for the delivery of ablative radiation doses while actively limiting the dose to the spinal cord and other areas at risk due to steep dose gradients (9). SBRT has shown to achieve durable pain relief, as well as high long-term local control rates independent of tumor histology and is subsequently increasingly being used to treat patients with spinal metastases (10, 11). The use of SBRT in the post-operative setting is, however, technically challenging. Precise planning and delivery of ablative radiation doses rely on accurate imaging including magnetic resonance imaging (MRI), and computed tomography (CT) examinations (9). Post-operatively, spinal implants cause imaging artifacts and prevent accurate delineation of the neural structures (9). Furthermore, radiation backscattering caused by spinal implants limits the biologically effective dose behind the implants, resulting in changed, and difficult to correct for, dosimetry (12, 13).

Reversing the order of surgery and SBRT could overcome the abovementioned technical challenges. Moreover, the ability with SBRT to actively limit the radiation dose to the posterior surgical area may eliminate the need for a time interval between the two treatment modalities. When both treatments would be administered within one hospital admission, SBRT induced pain relief could be experienced earlier, the treatment burden decreases and the start of adjuvant systemic therapies may be advanced.

Although both surgery and SBRT have proven to be safe and effective for the treatment of spinal metastases (14), the safety and feasibility of executing both modalities within a 24 h timeframe is yet unknown and was therefore investigated in this study.

## Methods

### Study Design and Patients

A non-randomized, single arm, single center, IDEAL stage I/IIa (see below) intervention study including patients with spinal metastases was conducted at the University Medical Center Utrecht, The Netherlands. Patients were eligible for inclusion if they were aged 18 years or older, had histological proof of malignancy, had symptomatic unstable spinal metastases in the thoracic or lumbar spine requiring surgery [based on clinical and imaging features, including the Spinal Instability Neoplastic Score (SINS) (15)], had a Karnofsky performance status of 50% or higher, and provided written informed consent. Patients were not eligible for inclusion if they had a diagnosis of a primary spinal bone tumor, had a history of prior radiation or surgery for the target spinal metastasis, required surgical stabilization of more than five adjacent spinal levels, had radiographic or symptomatic spinal cord compression [Bilsky 2 and 3 (16)], presented with rapidly deteriorating neurological deficits defined as the decline of one or more ASIA scale within 24 h, or had a life expectancy of < 3 months. An orthopedic surgeon and radiation oncologist together evaluated the eligibility of all patients. The local ethics board approved the study protocol.

This study was designed and conducted according to the IDEAL recommendations for the evaluation of complex surgical interventions (17). In stage I, the new treatment was used for the first time in three patients with a minimum time interval of 6 weeks between each patient to allow for identification of early major safety and/or feasibility issues. In stage IIa, 10 patients were enrolled to further evaluate the safety/feasibility of the new treatment strategy and allow for technical modifications if necessary.

### Study Procedures

**Figure 1:**
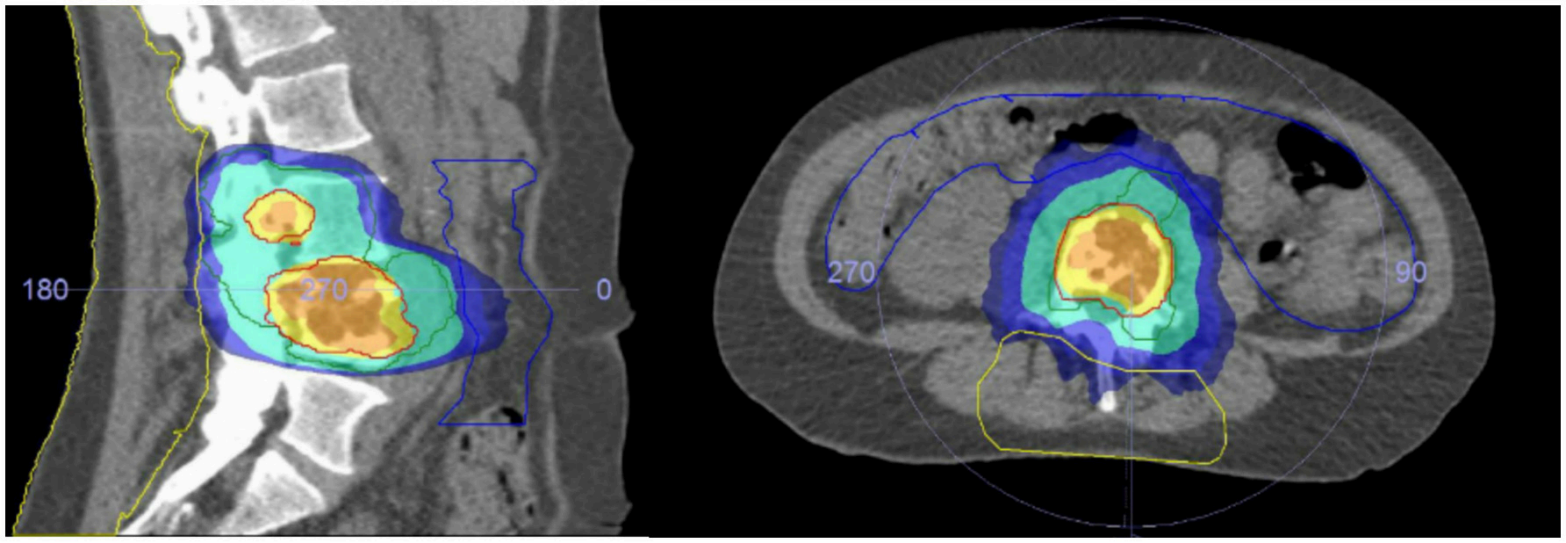
Planning CT showing a L4 metastases. The surgical area is depicted with the yellow lines. The dark blue area receives 8Gy, the turquoise area 9Gy, the yellow area 16.2Gy, and the orange area 18Gy.

After obtaining informed consent patients underwent a planning CT (Philips Medical Systems, Cleveland, OH) and 1.5 Tesla MRI scan (Ingenia; Philips Medical System, Best, The Netherlands) in SBRT treatment position. A single dose of 18Gy was prescribed to the macroscopic volume of the spinal metastasis. The bony compartment harboring the spinal metastasis was prescribed 8Gy to treat any subclinical disease. The macroscopic tumor volume, surrounding bony compartment, the organs at risk, and the posterior surgical area were delineated using MRI data. The posterior surgical area was delineated cranially and caudally by the lower, respectively, the upper endplate of the vertebral body adjacent to the upper and lower level of pedicle screw instrumentation; anteriorly by the contour of the vertebral body, laterally by the tips of the transverse processes, and posteriorly by the outermost layer of the skin (Figure 1). The dose to the surgical area was actively limited to as low as reasonable achievable during planning using a rotating beam technique. Dose constraints for the spinal cord (Max 12 Gy, V10Gy < 0.35cc) and other organs at risk were of primary concern while preparing the SBRT treatment plan and violations of these constraints were not accepted. The dose constraints for the OAR were based on local institutional guidelines. A detailed description of the radiotherapy planning is described elsewhere [(18), manuscript accepted Acta Oncologica]. Administration of dexamethasone was at the discretion of the treating radiation oncologist. SBRT treatment was delivered on a priority basis within 24 h prior to the planned surgical procedure.

The surgical technique, either a percutaneous (Longitude, Medtronic) or conventional open (Universal Spine System, Depuy Synthes) approach, was determined by the surgeon depending on the need for decompression of neurological structures and the spinal level(s) affected. If indicated, vertebral body stenting with poly methyl methacrylate was used to reinforce the anterior spinal column. Intraoperative and post-operative care was performed according to the local standard of care.

### Outcomes

The primary outcome of this study was safety of the combined procedure (SBRT and surgery with 24 h) within 90 days following treatment, with the occurrence of wound complications being the primary concern. Adverse events were evaluated and classified according to the Common Toxicity Criteria Adverse Events 4.0 (CT-CAE 4.0) during hospital stay on a daily basis by a researcher and during follow-up in the outpatient clinic. Wound healing complications were defined as grade 2 or higher treatment-induced toxicity according to CTC-AE 4.0. and included wound infections, wound dehiscence, radiation dermatitis, or soft tissue necrosis. The secondary outcomes were evaluated at baseline and 4, 8, 12 weeks post-treatment and included pain response measured with the Brief Pain Inventory (19) according to the International Bone Metastases Consensus Endpoints for Clinical Trials (20), length of hospital stay (days), neurological status as defined by the ASIA scale (21) and quality of life using the EQ-5D, the EORTC QLQ-C15-PAL, the EORTC QLQ-BM22, and the Spine Oncology Study Group Outcomes Questionnaire (SOSGOQ) (22). Patients were contacted by a researcher in case the questionnaires were not returned in time. Patients returned to routine clinical follow-up every 6 months after completion of the study.

### Statistical Analysis

A sample size of 13 patients was predefined based on the IDEAL recommendations for stage I and IIa. An independent data safety monitoring board (DSMB) consisting of content experts was established before the start of the study. Results were presented to the DSMB after the third and eight patients, stopping criteria were predefined based on the occurrence of wound complications. Descriptive statistics were used to describe demographic and treatment data, using RStudio (Version 0.99.903). This study was registered in the ClinicalTrials.Gov database (NCT02622841).

## Results

Fifteen patients with symptomatic unstable spinal metastases were recruited between June 2015 and January 2017. Two patients were excluded before SBRT treatment due to development of mild neurological deficits at the time of hospital admission and inconclusive preoperative pathology. Thirteen patients were treated according to the study protocol and underwent SBRT followed by surgical stabilization. The most common primary tumor was breast carcinoma followed by lung carcinoma and renal cell carcinoma (Table 1). For eight patients, painful spinal metastases were the first symptom of their primary tumor. At the time of surgery two patients had evidence of lymph node metastases and three patients had evidence of visceral and lymph node metastases.

**Table 1:** Baseline characteristics of treated patients.

**Age at time of surgery (range)**	**Primary tumor**	**Affected level**	**Karnofsky**	**SINS**	**Pain score**	**ASIA**
76–80	Renal cell	L2	60	9	4.0	E
41–45	Breast	L3	60	13	5.4	E
61–65	Lung	T9	90	9	3.4	E
56–60	Breast	T10	70	9	2.8	E
66–70	Prostate	L4	80	11	5.3	D
51–55	Breast	L3	80	9	4.6	E
56–60	Prostate	T1	60	12	4.7	E
61–65	Renal cell	T6	90	10	1.6	E
61–65	Melanoma	L4	90	7	5.7	E
41–45	Breast	T11	60	11	9.1	E
71–75	Lung	T9	70	10	1.2	E
41–45	Lung	T10	80	8	4.7	E
81–85	Renal cell	L3	70	10	5.3	E

All patients underwent surgery within 24 h after SBRT; the median time between SBRT and surgery was 17 h (IQR 5–19 h) with four patients receiving both treatments on the same day. All patients underwent single fraction SBRT with a mean dose to the target metastasis of 17.7Gy (range 16.4–18.6Gy) and a mean radiation dose of 2.9Gy (range 1.6–5.3Gy) to the surgical area (Table 2). Eleven patients underwent percutaneous pedicle screw fixation and two patients underwent an open procedure including decompression of neurological elements. The median operation time (incision until wound closure) was 68 min (IQR 60–90 min) with a median blood loss of 50 ml (range 50–300). All patients were able to ambulate on the first day after surgery. The median length of hospital stay was 5 days (IQR 4–6 days) measured from the day of SBRT until discharge.

**Table 2:** SBRT dosimetrics.

Mean dose to macroscopic tumor volume	17.7Gy (range 16.4–18.6Gy)
Mean coverage macroscopic tumor volume^*^	82% (range 67–93.4%)
Mean coverage of vertebral body^†^	99% (range 97–100%)
D1cc of the spinal cord^§^	7.8Gy (range 0.2–9.6Gy)
D1cc of the cauda^§^	12.5Gy (range 11.9–13Gy)
Mean dose to the surgical area	2.9Gy (range 1–5.3Gy)
Mean volume of surgical area (cc)	455 (range 243–888cc)

* *% of GTV that received 16.2Gy or higher*,

† *% of CTV that received 7.2Gy or higher*,

§ *maximum dose in Gy to 1cc of the volume*.

### Adverse Events

In the interval between SBRT and surgery we observed two grade 1 adverse events; nausea and radiation dermatitis (erythema). Cement leakage outside the vertebral body was observed intra-operatively with fluoroscopy in three patients. In one patient, this caused grade 3 radiculitis due to compression of the exiting left L3 nerve root requiring re-operation to decompress the root. The re-operation was performed 2 weeks after the initial treatment and resolved the complaints. Postoperatively, we observed the following grade 1–2 adverse events; nausea (1 event), diarrhea (2 events), constipation (2 events), transient urinary retention unrelated to cauda equina/spinal cord function (1 event), transient paresthesia of the leg (1 event), anemia not requiring transfusion (1 event) and transient radiculitis (1 event). One grade 3 syncope occurred post-operatively. None of the patients experienced disturbed wound healing or wound infection.

### Clinical Outcomes

At the time of study completion, the median clinical follow-up time was 13 months (IQR 10–17 months), one patient died due to systemic disease progression 14 months after the procedure. The mean BPI severity score at baseline was 3.8 (range 1–7) and decreased to 2.7 (range 0–5) at 4 weeks post-treatment, with all but one patient reporting a decrease in pain. All patients experienced a partial pain response during follow-up according to the consensus criteria. At 4 weeks post-treatment substantial improvements were reported in all domains of the SOSGOQ, BM-22, QLQ-C15, and the EQ-5D with further improvement over time (Table 3).

**Table 3:** Quality of life and pain scores over time.

	**Baseline (95%CI)**	**4 weeks (95%CI)**	**8 weeks (95%CI)**	**12 weeks (95%CI)**
**BPI**				
Intensity^*^	4.5 (3.2–5.7)	3.3 (2.0–4.7)	2.0 (0.8–3.2)	2.6 (1–4.2)
Severity^*^	3.8 (2.7–4.8)	2.8 (1.8–3.8)	2.4 (1.2–3.5)	2.9 (1.4–4.3)
**BM22**				
Painful Site^*^	29.2 (17.0–41.4)	20.0 (13.8–26.2)	16.4 (10.6–22.5)	21.1 (11.2–30.9)
Painful characteristics^*^	38.9 (24.9–52.8)	24.8 (14.1–35.45)	24.8 (13.1–35.4)	24.2 (13.8–34.7)
Social aspects	54.6 (41.4–68.1)	60.7 (47.3–74.0)	66.7 (56.8–76.5)	69.2 (57.9–80.4)
Functional Interference	56.4 (38.8–74.0)	71.9 (55.1–88.6)	76.4 (67.6–85.2)	75.0 (63.0–87.0)
**QLQ-C15**				
Pain^*^	56.9 (38.6–75.2)	38.5 (27.3–49.6)	29.2 (16.3–42.0)	22.2 (10.0–34.4)
Physical	45.6 (22.9–68.1)	66.7 (52.9–80.4)	76.4 (65.3–87.5)	78.9 (68.2–89.5)
Global	44.9 (25.9–63.9)	69.2 (60.2–78.3)	71.8 (61.4–82.2)	68.1 (55.7–80.4)
**SOSGOQ**				
Pain	48.1 (34.4–58.7)	64.2 (53.8–74.6)	69.2 (58.5–80.0)	77.1 (63.9–90.3)
Physical function	51.3 (31.6–70.9)	60.8 (51.3–70.2)	65.1 (56.0–74.1)	71.2 (59.5–82.8)
Social function	53.5 (46.2–60.7)	50.0 (40.4–59.6)	50.1 (41.1–60.2)	52.1 (43.9–60.3)
Mental health	65.4 (51.2–78.9)	70.2 (55.2–85.2)	75.0 (61.9–88.0)	77.1 (64.5–89.7)
EQ-5D	0.46 (0.25–0.65)	0.69 (0.6–0.8)	0.74 (0.64–0.82)	0.77 (0.68–86)

* *Decrease in score corresponds with improvement of symptoms*.

Thirteen months post-operatively one patient presented with neurological deficit based on recurrence of disease in the cranial level adjacent to the index level requiring emergency surgical decompression. In addition, the patient received post-operative palliative radiotherapy to T7-T11 using a 5x4Gy treatment schedule.

## Discussion

In this first-in-man study we demonstrated the safety and feasibility of single fraction SBRT, with active dose-limiting of the surgical area, followed by surgical stabilization within 24 h for the treatment of symptomatic unstable spinal metastases. Substantial improvements in pain and quality of life scores were observed for all patients over time. Minimal clinically important difference (MCID) values have previously been reported for the BM-22 and the QLQ-15 (23). The biggest improvements in quality of life were observed in the first 4 weeks following treatment with further improvement at 8 and 12 weeks post-treatment, improvements in all BM-22 domains and in the pain, physical and global quality of life domain of the QLQ-15 were greater than or equal to the previously reported MCID's.

This new treatment strategy has several advantages. First, with both procedures being performed within one hospital admission the treatment burden for the patient is substantially decreased. Second, by eliminating the time interval between treatments the radiation induced pain relief is experienced earlier as Ryu et al. demonstrated a median time to pain response after SBRT treatment of 14 days with a response achieved as early as 24 h after treatment (24). Moreover, complete response rates of up to 39% at 4 weeks post-SBRT treatment have been reported (24, 25). Third, pre-operative SBRT treatment planning is less challenging compared to post-operative treatment planning and delivery with its associated imaging artifacts and radiation scattering due to spinal implants. Furthermore, obtaining accurate imaging is physically demanding for the patient in the first days following surgery. Fourth, adjuvant therapies may be initiated earlier as these are often delayed until irradiation and initial wound healing is completed. Lastly, another potential advantage of pre-operative SBRT is the potential to decrease the spread of vital tumor cells due to surgical manipulation. Experimental animal studies have shown that single high doses of radiation (15-20Gy), as achieved with SBRT, result in tissue damage as early as 1–6 h after irradiation (26, 27). The vitality of tumor cells that are spilled into the bloodstream and neighboring tissues by surgical manipulation (28) and the subsequent potential acceleration of tumor spread and progression of disease, may therefore be reduced with reversing the order of surgery and SBRT.

We observed one serious adverse event, which was a grade 3 radiculitis requiring re-intervention. However, the DSMB regarded this as an isolated surgical incident secondary to cement extravasation rather than the result of combining SBRT with surgery. One patient developed neurological deficits 13 months post-SBRT based on recurrence of disease in the adjacent vertebra. This was in line with the study of Koyfman et al. reporting 12.5% recurrence in the adjacent level at a median time of 7.7 months after SBRT (29). Other adverse events were consistent with known reported adverse events associated with surgery and SBRT (9, 30).

One of the primary concerns of combining surgery and radiotherapy within a short timeframe is the occurrence of wound complications, which were not observed in any of our patients. The first phase of wound healing is particularly vulnerable for radiation exposure and with the use of conventional EBRT, the dose to the skin and underlying soft tissues is high (26, 27). Disturbed wound healing rates up to 46% have been reported when surgery and EBRT were performed within 1 week and a minimum time interval between surgery and EBRT of 1–2 weeks was therefore recommended (3, 31). The use of SBRT likely decreases the risk of wound complications as the conformal dose distribution allows for active sparing of healthy tissues overlying the surgical field. A recent systematic review investigated the effect of the timing of SBRT on the occurrence of wound complications (5). The evidence is limited to small observational studies and none of the studies considered wound complications as primary outcome (5). No time intervals of less than a week between surgery and SBRT were reported and considering the normal wound healing process an interval of at least 1 week was recommended (5).

Despite the conformal dose distribution, a distance between the spinal cord and tumor is necessary to deliver an ablative radiation dose while limiting the dose to the cord. The concept of separation surgery was therefore introduced (32). Tumor resection is limited to decompression of the spinal cord to allow for the use of post-operative SBRT to achieve local tumor control. Laufer et al. demonstrated in a series of 186 patients treated with separation surgery a 1-year local control rate of 90% to 96% depending on the SBRT fractionation schedule (32). Although these results are promising, it should be noted that SBRT was administered 2–4 weeks after surgery and an additional CT myelogram was required for accurate treatment planning increasing the treatment burden for the patient. The majority of 186 patients presented with cord compression [Bilsky 2 & 3 (16)] warranting separation of the tumor and the cord, but 25% of the patients presented with limited epidural disease, similar to our patients, and potentially could have been treated with the treatment strategy investigated in the current study.

We acknowledge the possible limitations of this study. Inherent to the study design, only a few and selected patients were included and subsequently the study is underpowered to detect any potential adverse advents with a low incidence. However, the main safety concern for the combination of radiotherapy and surgery within a short time frame is disturbed wound healing with substantial wound complication rates previously reported (3, 31). Furthermore, only patients without radiological or symptomatic spinal cord compression (Bilsky 1a-1c) were included as a distance between the spinal cord and the tumor is necessary for the safe delivery of an ablative dose and to avoid emergent treatment planning in this safety study. Lastly, although only one patient demonstrated a local recurrence 13 months after treatment, the true imaging-based local control rate for all patients is unknown. Patients were followed clinically, including routine follow-up imaging of the spine, but without specific imaging for the early detection of local recurrence.

In conclusion, this study demonstrated the safety and feasibility of SBRT, with active sparing of the surgical area, followed by surgical stabilization within 24 h for the treatment of symptomatic unstable spinal metastases with none of the patients demonstrating disturbed wound healing. Combining the two treatments within 24 h decreases the treatment burden for the patient, as no return visits for radiotherapy are necessary, and may result in earlier and improved pain response and local control rates compared to the current standard of care of surgery followed by EBRT. An IDEAL stage IIb study is currently planned to evaluate the effectiveness of the new treatment strategy and to obtain additional data to potentially change the standard of care for patients with symptomatic unstable spinal metastases.

## Author Contributions

AV, JvdV, JH, WE, NK, HV, FO, ES, and J-JV all had a substantial contribution to the conception or design of the work; drafting the work or revising it critically for important intellectual content and final approval of the manuscript to be published.

### Conflict of Interest Statement

The authors declare that the research was conducted in the absence of any commercial or financial relationships that could be construed as a potential conflict of interest.
